# Coronin 1 Is Required for Integrin β2 Translocation in Platelets

**DOI:** 10.3390/ijms21010356

**Published:** 2020-01-05

**Authors:** David R. J. Riley, Jawad S. Khalil, Jean Pieters, Khalid M. Naseem, Francisco Rivero

**Affiliations:** 1 Centre for Atherothrombosis and Metabolic Disease, Hull York Medical School, Faculty of Health Sciences, University of Hull, Hull HU6 7RX, UK; David.riley@hyms.ac.uk (D.R.J.R.); jawad.khalil@bristol.ac.uk (J.S.K.); 2 School of Physiology, Pharmacology and Neuroscience, Faculty of Life Sciences, University of Bristol, Bristol BS8 1TD, UK; 3 Biozentrum, University of Basel, CH-4056 Basel, Switzerland; jean.pieters@unibas.ch; 4 Leeds Institute for Cardiovascular and Metabolic Medicine, University of Leeds, Leeds LS2 9NL, UK; k.naseem@leeds.ac.uk

**Keywords:** actin, Arp2/3 complex, cAMP, coronin 1, integrin β2, platelets, thrombin, collagen, prostacyclin

## Abstract

Remodeling of the actin cytoskeleton is one of the critical events that allows platelets to undergo morphological and functional changes in response to receptor-mediated signaling cascades. Coronins are a family of evolutionarily conserved proteins implicated in the regulation of the actin cytoskeleton, represented by the abundant coronins 1, 2, and 3 and the less abundant coronin 7 in platelets, but their functions in these cells are poorly understood. A recent report revealed impaired agonist-induced actin polymerization and cofilin phosphoregulation and altered thrombus formation in vivo as salient phenotypes in the absence of an overt hemostasis defect in vivo in a knockout mouse model of coronin 1. Here we show that the absence of coronin 1 is associated with impaired translocation of integrin β2 to the platelet surface upon stimulation with thrombin while morphological and functional alterations, including defects in Arp2/3 complex localization and cAMP-dependent signaling, are absent. Our results suggest a large extent of functional overlap among coronins 1, 2, and 3 in platelets, while aspects like integrin β2 translocation are specifically or predominantly dependent on coronin 1.

## 1. Introduction

Vascular injury leads to exposure of prothrombotic extracellular matrix proteins, which facilitates the entrapment and activation of platelets through specialized receptors. These interactions contribute to stable adhesion of platelets by generating intracellular signals that lead to shape change, secretion of granules, and activation of integrins. Activation of integrins facilitates the binding of the plasma protein fibrinogen, which subsequently supports platelet aggregation and clot formation, rapidly consolidated by secreted soluble agonists [1]. While this process is critical to hemostatic protection of the vasculature after injury, the rupture of atherosclerotic plaques drives uncontrolled platelet activation that leads to arterial thrombosis and clinical events such as myocardial infarction and stroke.

Platelet activation is the result of multiple integrated signaling cascades that ultimately drive remodeling of the platelet cytoskeleton and sustain the morphological changes required for adhesion, spreading, aggregation, and secretion at the sites of vascular damage [2]. The cytoskeleton is also the target of inhibitory signaling pathways regulated by cyclic nucleotides that balance the activating pathways and prevent thrombus formation [3]. Coronins are a family of evolutionarily conserved regulators of the actin cytoskeleton turnover represented by seven members in mammals They have been grouped into three classes based on phylogenetic and functional criteria [4,5]. Class I includes Coronins 1, 2, 3, and 6 (also called 1A, 1B, 1C, and 1D) that associate with the actin cytoskeleton, localize at the leading edge of migrating cells, and participate in various signaling processes. Class II includes Coro4 and 5 (also called 2A and 2B), involved in focal adhesion turnover, reorganization of the cytoskeleton, and cell migration. The class III coronin (Coro7) has an unusual structure and plays a role in Golgi morphology maintenance. We have reported that class I coronins coronin 1, 2, and 3 are abundant in both human and mouse platelets, whereas coronin 7 is also present in human and mouse platelets in very low amounts and class II coronins are apparently absent [6].

Coronin 1 (coronin-1A or Coro1, also known as P57 or Tryptophan Aspartate containing COat protein (TACO)) [7,8] participates in the modulation of a number of processes through protein–protein interactions. For example, it modulates cyclic adenosine monophosphate (cAMP) signaling in neurons through interaction with the Gαs subunit of heterotrimeric G proteins [9], neutrophil adhesion through interaction with the cytoplasmic tail of integrin β2 [10], and the activity of the small GTPase Rac1 [11]. Coro1 also participates in a number of other cellular processes including NADPH oxidase complex regulation, calcium signaling, vesicle trafficking, and apoptosis [12,13,14,15,16].

Coro1 is abundantly expressed in cells of the hematopoietic lineage, where it is essential for the survival of naïve T cells [16,17,18,19], but little is known about its role in platelets. We have shown that Coro1 is mainly a cytosolic protein, but a significant amount associates to membranes in an actin-independent manner. It rapidly translocates to the detergent-insoluble cytoskeleton upon platelet stimulation with thrombin or collagen. Along with Coro2 and 3, it accumulates at the cell cortex and actin nodules [6]. Stocker et al. reported the absence of an overt hemostasis defect in vivo in a knockout mouse model of Coro1. Detailed examination revealed impaired agonist-induced actin polymerization and cofilin phosphoregulation and altered thrombus formation in vivo as salient phenotypes [20]. Here we extend Stocker et al. report by an in-depth characterization of platelet function exploring additional aspects. Our data show that the absence of Coro1 is associated with impaired translocation of integrin β2 to the platelet surface upon stimulation with thrombin but otherwise does not result in noticeable morphological and functional alterations, including Arp2/3 complex localization and cAMP-dependent signaling. This mild phenotype suggests a complex picture in which class I coronins might share roles extensively in platelets.

## 2. Results

### 2.1. Absence of Coro1 Is Not Compensated by Increased Coro3

**Figure 1:**
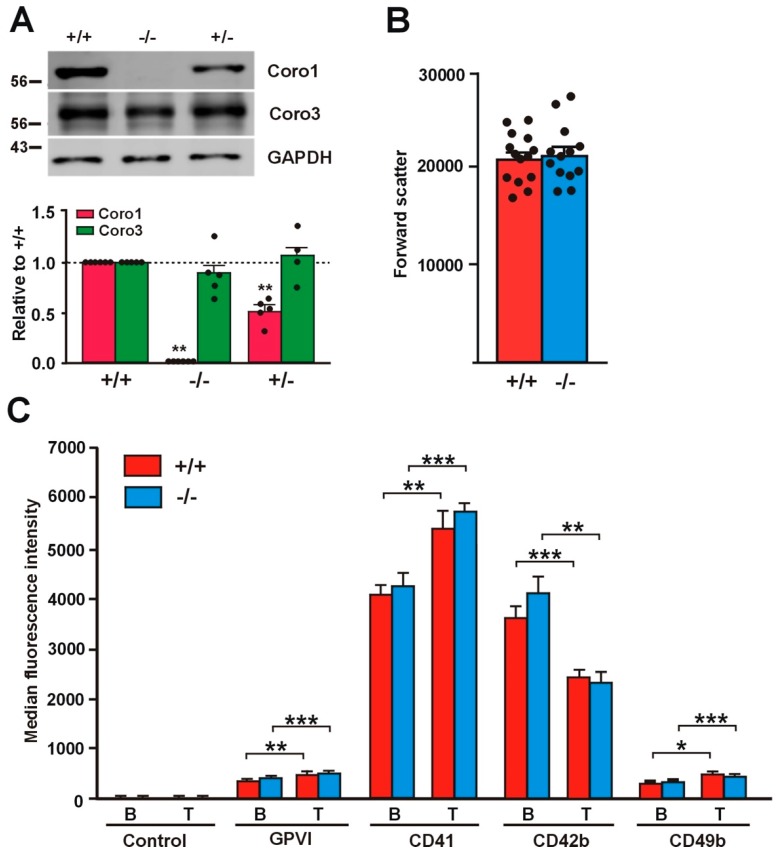
Relative size and receptor expression in *Coro1a* deficient platelets. (**A**) Absence of Coro1 in *Coro1a* deficient platelets and no obvious compensation by Coro3. Platelet lysates were resolved by SDS-PAGE, blotted and probed with specific antibodies for the indicated proteins. GAPDH was used for normalization. Data represent mean ± standard error of the mean (SEM) of 4–6 independent experiments. ** *p* < 0.01; Mann–Whitney U-test. Full blots are shown in Supplemental Figure S1; (**B**) Relative size of *Coro1a* deficient platelets. Mean platelet volume was estimated in platelet-rich plasma (PRP) by mean forward light scatter area using flow cytometry. Data represent mean ± SEM of 13–14 independent experiments. No statistically significant differences were found, Student’s *t*-test; (**C**) Surface receptor expression in *Coro1a* deficient platelets. Platelet surface receptors were determined in PRP by flow cytometry both in basal conditions (B) and upon stimulation with 0.1 U/mL thrombin for 20 min at 37 °C (T). Data represent mean ± SEM of 7–16 independent experiments. * *p* < 0.05; ** *p* < 0.01; *** *p* < 0.001; paired Student’s *t*-test between basal and stimulated conditions. No statistically significant differences were found between wild type and knockout, nonpaired Student’s *t*-test.

To gain insight into the roles of Coro1 in platelet function, we undertook the characterization of a previously described *Coro1a* knockout (KO) model [15]. We confirmed the absence of the protein in platelet lysates of homozygous KO mice by Western blot analysis and observed that heterozygous mouse platelets expressed approximately half of the amount of the protein present in wild type (WT) mouse platelets (Figure 1A). Coro1 KO mice have been reported to exhibit unaffected hematological parameters, including platelet counts, indicating that hematopoiesis is not affected [17,20]. The size of Coro1 KO platelets was comparable to that of WT platelets as estimated from the forward light scatter in flow cytometry experiments (*p* = 0.8164, Student’s *t*-test) (Figure 1B).

### 2.2. Receptor Expression Is Not Affected in Coro1 Deficient Platelets

We assessed the expression of characteristic surface platelet receptors (GPVI, CD41, CD42b, and CD49b) by flow cytometry both in unstimulated and in thrombin-stimulated platelets. Thrombin stimulation caused a significant increase in the expression of GPVI, CD41 (integrin αIIb), and CD49b (integrin α2) (20–40%) and a significant decrease in the expression of CD42b (GP1b) (32–43%), the latter due to cleavage and internalization of the GP1b/IX/V complex [21]. Both basal and thrombin-stimulated receptor expression levels were comparable in Coro1 WT and KO platelets (Figure 1C).

### 2.3. Translocation of Integrin β2 Is Impaired in the Absence of Coro1

**Figure 2:**
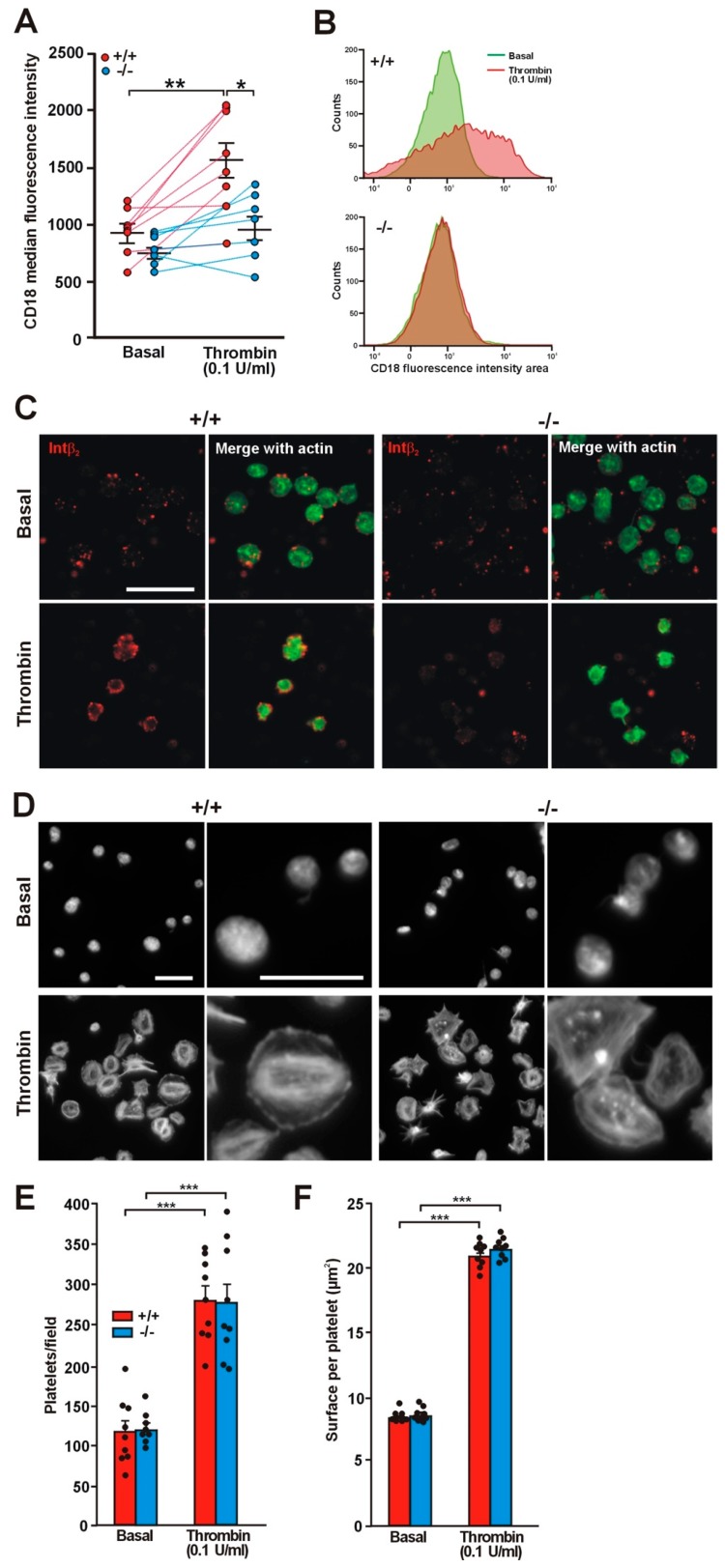
Impaired translocation of integrin β2 in *Coro1a* deficient platelets. (**A**) Platelet surface integrin β2 (CD18) was determined in PRP by flow cytometry both in basal conditions and upon stimulation with 0.1 U/mL thrombin for 20 min at 37 °C. Individual data and the mean ± SEM of 7–8 independent experiments are shown. * *p* < 0.05; ** *p* < 0.01; paired Student’s *t*-test between basal and stimulated conditions. Nonpaired Student’s *t*-test between wild type (WT) and knockout (KO); (**B**) Representative flow cytometry data of platelet surface CD18 distribution in basal conditions and upon thrombin stimulation; (**C**) Washed platelets were stimulated in suspension with 0.1 U/mL thrombin, fixed with 4% paraformaldehyde (PFA) and spun on poly-l-lysine coated coverslips. The permeabilization step was omitted and the cells were stained with an anti-integrin β2 antibody followed by an Alexa568-coupled secondary antibody (red) and counterstained with fluorescein isothiocyanate (FITC)-phalloidin for filamentous actin (green). Images were acquired with a fluorescence microscope equipped with a structured illumination attachment and deconvolved. Scale bar represents 10 μm; (**D**) Adhesion of Coro1 KO and WT platelets to native bovine serum albumin (BSA). Washed platelets were stimulated with 0.1 U/mL thrombin and immediately allowed to attach to glass coverslips coated with 5 mg/mL of native BSA. Adherent platelets were fixed with 4% PFA, permeabilized with 0.3% Triton X-100, and stained with tetramethylrhodamine isothiocyanate (TRITC)-phalloidin. Images of random areas were acquired with a fluorescence microscope. Examples of platelets at two magnifications are shown. Scale bars represent 10 μm; (**E**) Number of platelets adhering to BSA. 5 fields each 31,560 μm^2^ from 9 independent experiments were scored per condition. Data represent mean ± SEM. Number of platelets was significantly higher upon thrombin stimulation (** *p* < 0.01, paired Student’s *t*-test). No significant differences were found between WT and KO platelets both resting and stimulated (unpaired Student’s *t*-test); (**F**) Surface coverage per platelet calculated by thresholding using ImageJ. Data represent mean ± SEM from 9 independent experiments and 600–1200 platelets per condition for each experiment. Platelet surface was significantly higher upon thrombin stimulation (*** *p* < 0.001, paired Student’s *t*-test). No significant differences were found between WT and KO platelets, both resting and stimulated (unpaired Student’s *t*-test).

Coro1 interacts with the cytoplasmic tail of integrin β2 and regulates its function in neutrophils [10]. Although less abundant than integrins β1 and β3, integrin β2 (CD18) is expressed in murine platelets [22,23,24,25] and has also been described in human platelets, where expression increases upon thrombin stimulation [26]. This prompted us to investigate whether Coro1 deficiency would have an effect on this integrin. We used flow cytometry to assess the levels of expression of CD18 both in resting and in thrombin stimulated platelets and observed that in resting platelets the levels of CD18 were higher, although statistically not significant, in WT platelets (940 ± 70 median fluorescence intensity) than in KO platelets (783 ± 51; *p* = 0.1016). However, upon thrombin stimulation expression increased significantly in WT platelets to 1562 ± 158 (*p* = 0.0032 relative to basal) but only modestly in KO platelets (to 986 ± 110; *p* = 0.0915 relative to basal, *p* = 0.0123 relative to WT) (Figure 2A,B). The impaired translocation of CD18 in KO platelets can be visualized in immunostained platelets (Figure 2C).

Integrin β2 main ligand is intercellular adhesion molecule-1 (ICAM-1), a glycoprotein expressed in endothelial cells and leukocytes. We used fluorescence microscopy to investigate the effect of Coro1 absence on platelet adhesion and spreading on surfaces coated with 5 mg/mL native BSA, a surrogate method of assessing binding to ICAM-1, both basally and upon stimulation with 0.1 U/mL thrombin [27,28]. On average, similar numbers of WT and KO resting platelets adhered to coverslips (116.7 ± 14.0 and 120.8 ± 7.1, respectively). Resting platelets of both strains attached to the BSA-coated surface but most did not appear to spread, presenting a round morphology and covering a small area (approximately 9 µm^2^) (Figure 2D–F). Thrombin stimulation prior to seeding resulted in more than twice the numbers of adhering platelets (280.3 ± 17.2 in WT vs. 276.9 ± 24.0 in KO). Most stimulated platelets presented a well spread round morphology with stress fibers, although some had a spiky morphology, and covered an area of approximately 21 µm^2^. No obvious differences were apparent in cell area between WT and KO platelets (Figure 2D–F). To investigate whether stimulation with lower thrombin doses would reveal any subtle difference in spreading between WT and KO platelets, we performed a set of experiments basally and upon stimulation with 0.05 and 0.025 U/mL thrombin. We observed that both doses resulted in numbers of adhering platelets similar to those obtained with 0.1 U/mL: 309.3 ± 16.8 in the WT vs. 282.0 ± 13.7 in the KO with 0.05 U/mL and 296.3 ± 22.4 in the WT vs. 320.8 ± 38.9 in the KO with 0.025 U/mL. The areas of the spread platelets were also in a range similar (20–21 µm^2^) to those observed with 0.1 U/mL thrombin. This indicates that low thrombin doses (0.025 U/mL) are sufficient to elicit full spreading on native BSA and Coro1 is dispensable for this response. 

### 2.4. Effect of Coro1 Deficiency on Integrin αIIbβ3 Activation and Granule Secretion

**Figure 3:**
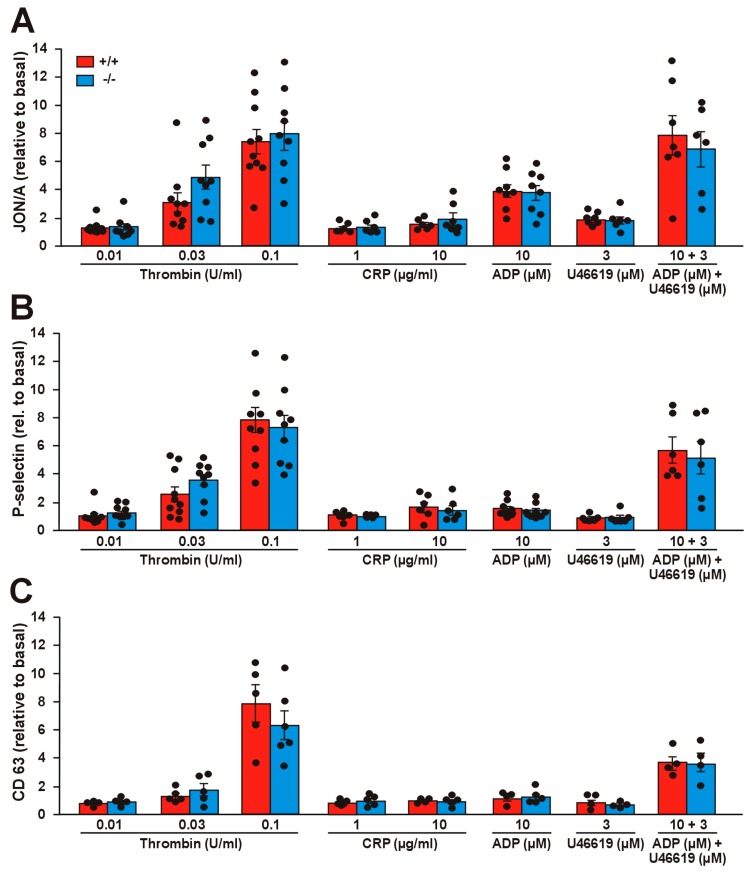
Integrin activation and secretion in *Coro1a* deficient platelets. Integrin activation (**A**), P-selectin exposure (**B**), and CD63 exposure (**C**) were determined in PRP upon stimulation with the indicated doses of agonists for 20 min at 37 °C and subsequent flow cytometry analysis. The data (median fluorescence intensity) represent the mean ± SEM of 5–9 independent experiments expressed relative to basal (unstimulated) platelets. No statistically significant differences were found between WT and KO, Student’s *t*-test.

We assessed the potential effects of Coro1 deficiency on integrin αIIbβ3 activation with the activation state-specific antibody JON/A by flow cytometry. Stimulation with a wide range of agonists (thrombin, collagen-related peptide (CRP), as well as adenosine diphosphate (ADP) and the thromboxane analog U46619 alone or in combination) caused activation of αIIbβ3, in the case of thrombin and CRP in a dose-dependent manner (Figure 3A). However, we were not able to detect any significant differences in JON/A levels between Coro1 KO and WT platelets, indicating that Coro1 is dispensable for αIIbβ3 activation. 

We next explored whether Coro1 KO platelets have a defect in granule secretion. To monitor alpha and dense granule secretion, we induced P-selectin and CD63 expression, respectively, by the same agonists as in the αIIbβ3 activation experiment. In both cases, thrombin produced a clear dose–response effect, CRP had little effect and ADP and U46619 had a synergistic effect in both WT and KO platelets (Figure 3B,C). None of the conditions tested revealed any statistically significant difference between both populations, suggesting that Coro1 is dispensable for granule secretion.

### 2.5. Effect of Coro1 Deficiency on Platelet Aggregation and Spreading

**Figure 4:**
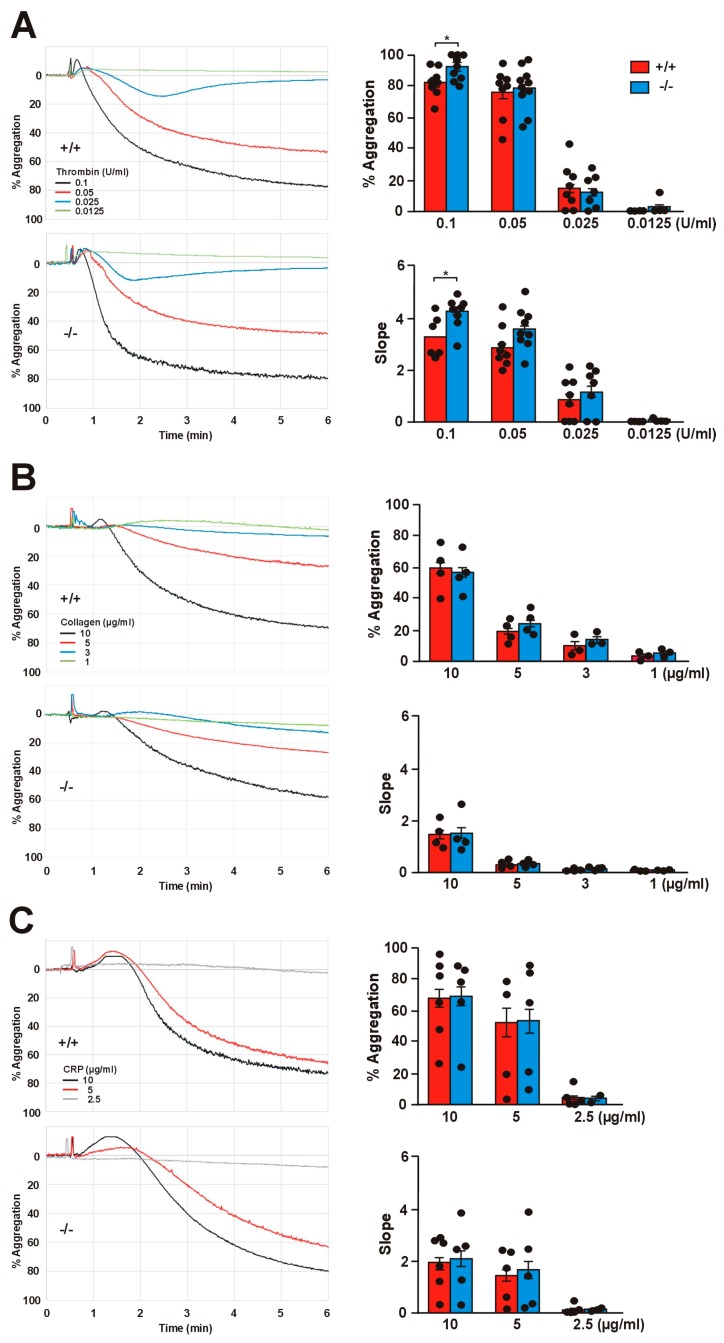
Aggregation in *Coro1a* deficient platelets. Washed platelets (2.0 × 10^8^ platelets/mL) were stimulated with the indicated doses of thrombin (**A**), collagen (**B**), or collagen-related peptide (CRP) (**C**) and aggregation was recorded for 6 min in a Chrono-Log aggregometer. Representative traces are shown on the left. Bar diagrams show percentage of maximum aggregation within 5 min of stimulation and slope as calculated from the linear part of the aggregation trace. Data are mean ± SEM of 4–10 independent experiments. * *p* < 0.05, Student’s *t*-test for thrombin; no significant differences were found with collagen and CRP, Mann–Whitney U-test.

A functioning actin cytoskeleton remodeling is critical for platelet aggregation and for adhesion and spreading on extracellular matrix proteins. We next investigated the implications of Coro1 deficiency for those processes. Stocker et al. reported subtle defects in aggregation induced by low doses of collagen using impedance-based aggregometry on whole blood [20]. We applied light transmission aggregometry on washed platelets using a range of doses of thrombin (0.0125–0.1 U/mL), collagen (1–10 µg/mL), and CRP (3–10 µg/mL). All three agonists elicited, as expected, a dose-dependent aggregation response, which was comparable in both WT and KO platelets at all doses (Figure 4). The aggregation velocity, calculated as the slope of the aggregation curve, was also dose-dependent for all three agonists. We only observed a statistically significant alteration in the response to high-dose thrombin, with KO platelets showing a marginally higher percentage of aggregation (91.7 vs. 82.4, *p* = 0.0420) and a moderately higher velocity (3.29 vs. 4.30, *p* = 0.0137, Student’s *t*-test) compared to WT platelets. We did not observe any statistically significant difference between WT and KO platelets at any dose of collagen or CRP.

**Figure 5:**
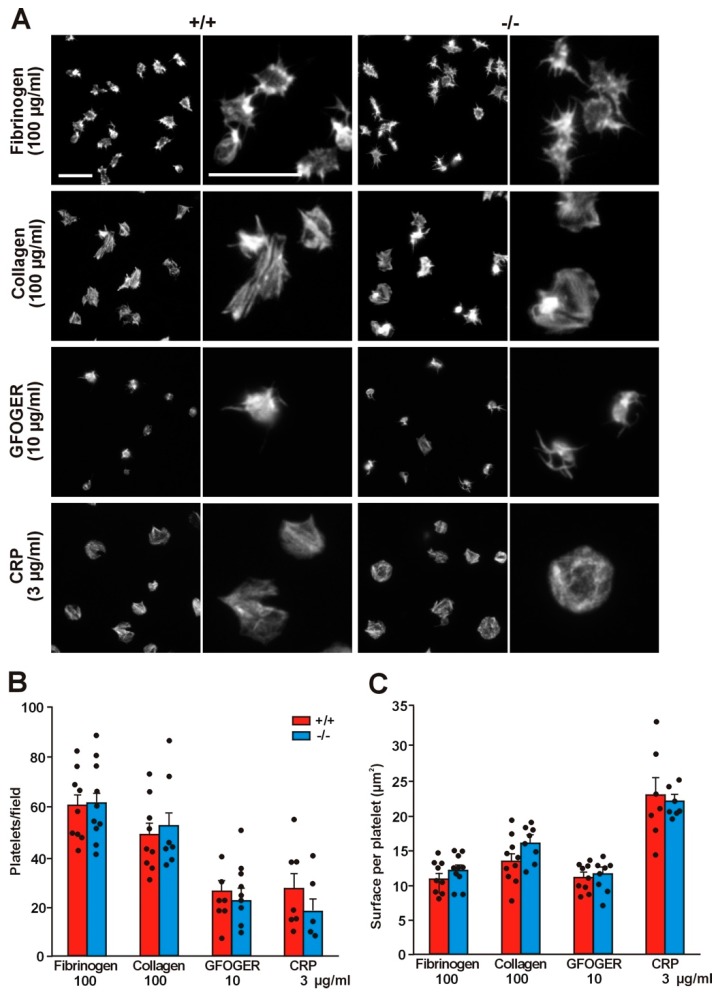
Absence of Coro1 does not impair platelet spreading. (**A**) Adhesion of washed platelets to glass coverslips coated with the indicated concentration of collagen, fibrinogen, Gly-Phe-Hyp-Gly-Glu-Arg (GFOGER), or CRP. Adherent platelets were fixed with 4% PFA, permeabilized with 0.3% Triton X-100, and stained with TRITC-phalloidin. Images were acquired with a fluorescence microscope equipped with a structured illumination attachment and deconvolved. Examples of platelets at two magnifications are shown. Scale bars represent 10 μm; (**B**) Number of platelets adhering to the indicated concentrations of collagen, fibrinogen, GFOGER, or CRP. 5 fields each 12,500 μm^2^ from 5–10 independent experiments were scored per condition. Data represent mean ± SEM. No significant differences were found between WT and KO platelets for any condition (Mann-Whitney U-test); (**C**) Surface coverage per platelet calculated by thresholding using ImageJ. Data represent mean ± SEM from 5–10 independent experiments and 250–1000 platelets per condition for each experiment. No significant differences were found between WT and KO platelets for any condition (Mann–Whitney U-test).

The effect of Coro1 absence on platelet adhesion and spreading was further investigated on surfaces coated with collagen (100 μg/mL) or fibrinogen (100 μg/mL) by fluorescence microscopy. On average, slightly more platelets per observation field adhered on fibrinogen; however, there were no statistically significant differences in the numbers of platelets adhering to either surface between the WT and the KO platelets (60.4 ± 4.5 vs. 61.4 ± 5.1 on fibrinogen and 48.3 ± 4.6 vs. 52.3 ± 7.4 on collagen) (Figure 5A, B). Irrespective of genotype, platelets covered a slightly larger surface on collagen (13.43 ± 1.07 µm^2^ in the WT vs. 15.83 ± 0.85 µm^2^ in the KO) than on fibrinogen (10.75 ± 0.74 µm^2^ in the WT vs. 11.89 ± 0.71 µm^2^ in the KO) (Figure 5C). Characteristically, on fibrinogen, most platelets showed abundant filopods and actin nodules whereas on collagen most displayed stress fibers, however, no differences in the morphology were apparent between WT and KO platelets in any of the matrices.

We investigated any subtle effect of Coro1 ablation on adhesion to specific collagen receptors using coverslips coated with peptides that discriminate between receptors, Gly-Phe-Hyp-Gly-Glu-Arg (GFOGER) (for α2β1 integrin), or CRP (for GPVI). Approximately 50% less platelets adhered on GFOGER and CRP compared to collagen and the trend was similar in both WT and KO platelets (Figure 5B). Surface coverage was lower on GFOGER (10.98 ± 0.62 µm^2^ in the WT vs. 11.43 ± 0.81 µm^2^ in the KO) and higher on CRP (22.92 ± 2.24 µm^2^ in the WT vs. KO 21.96 ± 0.77 µm^2^ in the KO) compared to collagen (Figure 5C). On CRP, platelets morphologically resembled the ones on collagen, whereas on GFOGER they were discoid and spiky. Again, there were neither statistically significant differences in surface coverage between WT and KO platelets nor noticeable morphological differences in collagen receptor-specific matrices.

### 2.6. Coro1 Is Dispensable for Arp2/3 Complex Localization

**Figure 6:**
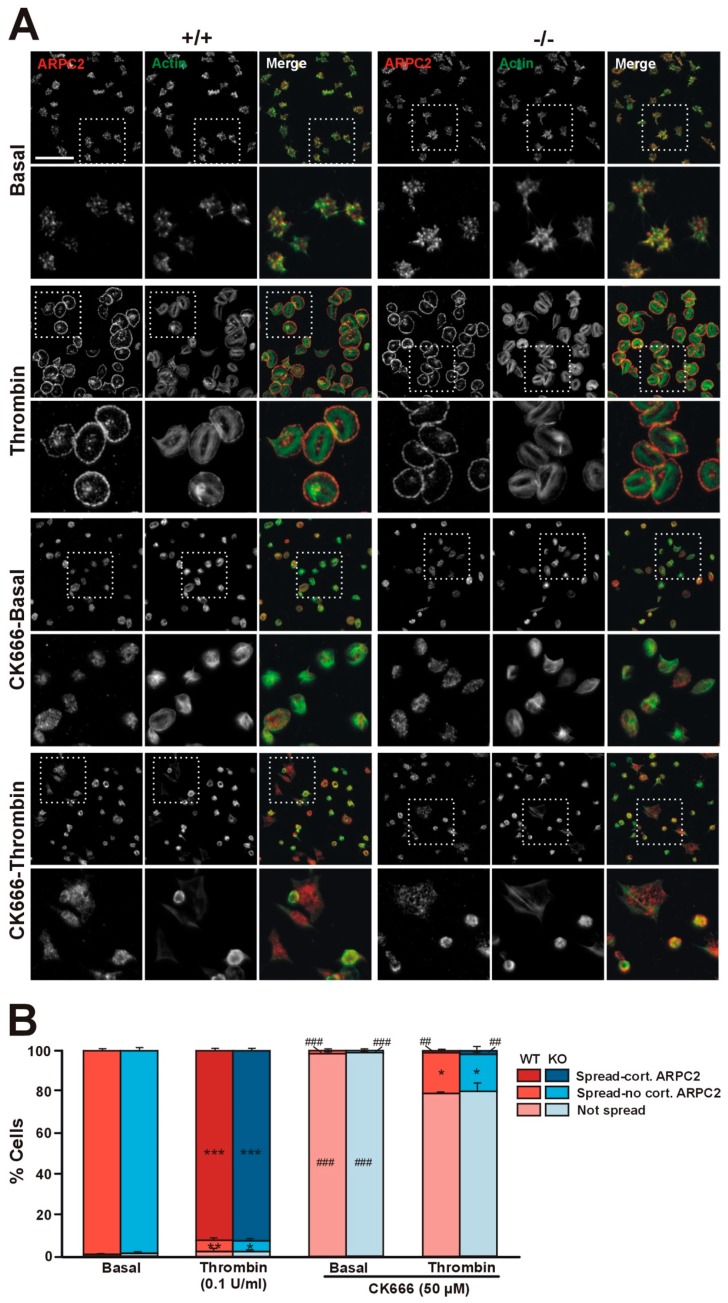
Coro1 is dispensable for Arp2/3 complex localization. (**A**) Localization of ARPC2 in resting and thrombin-stimulated platelets. Washed platelets were stimulated with 0.1 U/mL thrombin and immediately allowed to attach to glass coverslips coated with 100 µg/mL of fibrinogen. A population of platelets was treated with the Arp2/3 complex inhibitor CK666 (50 µM) for 30 min at 37 °C prior to thrombin stimulation. Adherent platelets were fixed with 4% PFA, permeabilized with 0.3% Triton X-100, stained with an anti-ARPC2 antibody followed by an Alexa568-coupled secondary antibody (red) and counterstained with FITC-phalloidin for filamentous actin (green). Images were acquired with a fluorescence microscope equipped with a structured illumination attachment and deconvolved. Examples of platelets at two magnifications are shown. Boxes mark the enlarged regions. Scale bar represents 25 μm; boxes are 27 × 27 µm; (**B**) Platelet morphology. Platelets were assigned to one of three classes based on spreading and ARPC2 distribution (cortical or not). 5 fields each 36,670 μm^2^ from 4 independent experiments were scored per condition. Data are shown as percentage of platelets of each class and represent mean ± SEM. * *p* < 0.05, ** *p* < 0.01, *** *p* < 0.001 relative to the corresponding basal condition. ^##^ *p* < 0.01, ^###^ *p* < 0.001 relative to platelets not treated with CK666 of the same condition. Symbols are placed inside their corresponding bars. No significant differences were found between WT and KO platelets for any condition (Kruskal–Wallis test).

We have shown that in platelets Coro1 co-immunoprecipitates and colocalizes with components of the Arp2/3 complex and that the activity of the complex is necessary for extension of lamellipodia and accumulation of Coro1 at the cell cortex of spreading platelets [6]. We used Coro1 deficient platelets to address whether Coro1 is necessary for Arp2/3 complex localization by allowing them to spread on fibrinogen upon activation with 0.1 U/mL thrombin (Figure 6A). Virtually all unstimulated platelets spread on fibrinogen and showed abundant filopods and actin nodules. In those platelets, the Arp2/3 component ARPC2 (p34-Arc, component of the Arp2/3 complex) displayed a diffuse distribution and some accumulation at actin nodules. Upon thrombin stimulation, approximately 92% of platelets adopted a well spread circular shape with stress fibers and neat accumulation of actin and ARPC2 at the cell cortex. These patterns of platelet morphology and ARPC2 distribution were indistinguishable in both WT and KO platelets (Figure 6B), indicating that Coro1 is dispensable for Arp2/3 localization and lamellipodia formation upon platelet stimulation. Using the same approach, we explored whether activation of the Arp2/3 complex is required for spreading and for cortical localization of ARPC2 upon thrombin stimulation (Figure 6A). We treated wild type and Coro1 deficient platelets with the Arp2/3 complex inhibitor CK666 (50 µM) prior to stimulation with thrombin and seeding. While still capable of adhering to fibrinogen, virtually all unstimulated platelets remained discoid with inconspicuous ARPC2 distribution. Upon thrombin stimulation, approximately 80% of platelets remained discoid, whereas the rest spread, however, their morphology was irregular, their F-actin staining was weaker compared to untreated platelets, and ARPC2 was almost never found at the cell cortex. This shows that activation of the Arp2/3 complex is required for its cortical localization and for efficient spreading, irrespective of the presence of coronin 1.

### 2.7. Absence of Coro1 Does Not Affect cAMP Signaling

**Figure 7:**
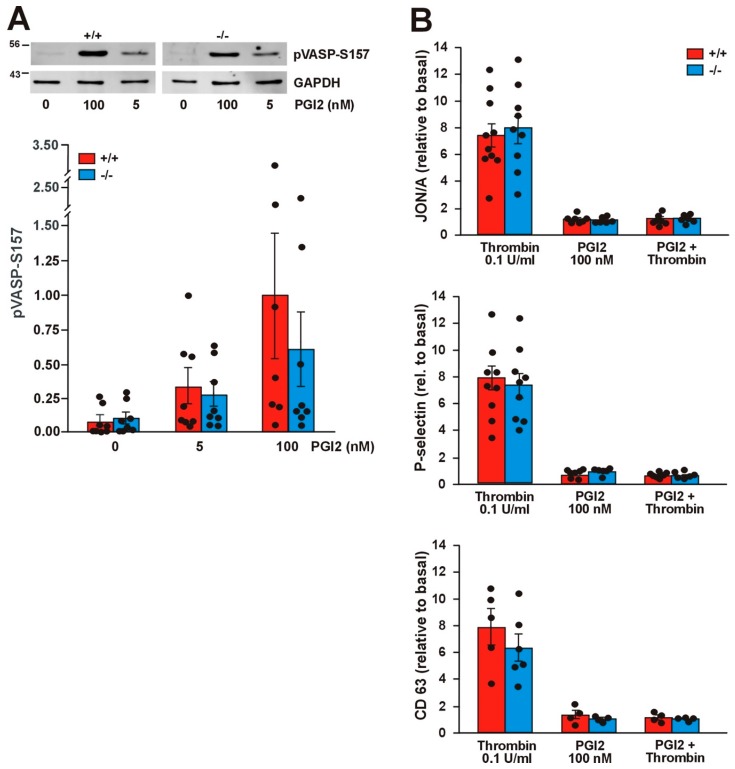
The cAMP pathway is not affected in *Coro1a* deficient platelets. (**A**) Phosphorylation of vasodilator-stimulated phosphoprotein (VASP) upon prostacyclin (PGI2) stimulation for 5 min at the indicated doses. Platelet lysates were resolved by SDS-PAGE, blotted, and probed with specific antibodies for pVASP-Ser157. GAPDH was used for normalization. Representative blots and bar diagrams showing mean ± SEM of 7 independent experiments. Full blots are shown in Supplemental Figure S2; (**B**) Integrin activation, P-selectin exposure, and CD63 exposure upon stimulation with thrombin prior to PGI2. Platelets in PRP were treated with 100 nM PGI2 for 5 min prior to stimulation with 0.1 U/mL thrombin and subsequently analyzed by flow cytometry. This set of experiments was carried out simultaneously with the ones presented in Figure 3. The data (median fluorescence intensity) are expressed relative to basal (unstimulated) platelets. The data represent the mean ± SEM of 4–9 independent experiments. No significant differences were found between WT and KO in any of the assays (Student’s *t*-test or Mann–Whitney U-test).

Jayachandran et al. have shown that Coro1 interacts with Gαs and modulates the cAMP signaling pathway in neurons and T cells [9,29]. In platelets the cAMP pathway can be triggered by exposure to prostacyclin (PGI2), whose receptor is coupled to heterotrimeric G proteins containing the Gαs subunit, resulting in dampening of the ability to respond to thrombin stimulation. We have shown that in platelets Coro1 is able to immunoprecipitate and colocalize with Gαs [6], prompting us to investigate the functionality of the cAMP pathway in Coro1 deficient platelets. We monitored the activity of the cAMP pathway by detection of vasodilator-stimulated phosphoprotein (VASP) phosphorylation at Ser157. Treatment with a low (5 nM) and a high (100 nM) dose of PGI2 resulted in a dose-dependent increase in the amount of pVASP-S157 in both WT and KO platelets. No statistically significant differences were observed between both genotypes (Figure 7A). We used flow cytometry to quantify the effect of PGI2 on thrombin-stimulated integrin αIIbβ3 activation and granule secretion. Platelets were pretreated with 100 nM PGI2 prior to stimulation with 0.1 U/mL thrombin. As already shown in Figure 3, stimulation with thrombin caused activation of integrin β3 as well as P-selectin and CD63 expression, whereas PGI2 itself did not elicit any response. Treatment with 100 nM PGI2 prior to thrombin stimulation completely abolished those responses both in WT and KO platelets (Figure 7B). Collectively, our results indicate that Coro1 is dispensable for Gαs-dependent modulation of the cAMP pathway in platelets.

### 2.8. Absence of Coro1 Does Not Impair Hemostasis

**Figure 8:**
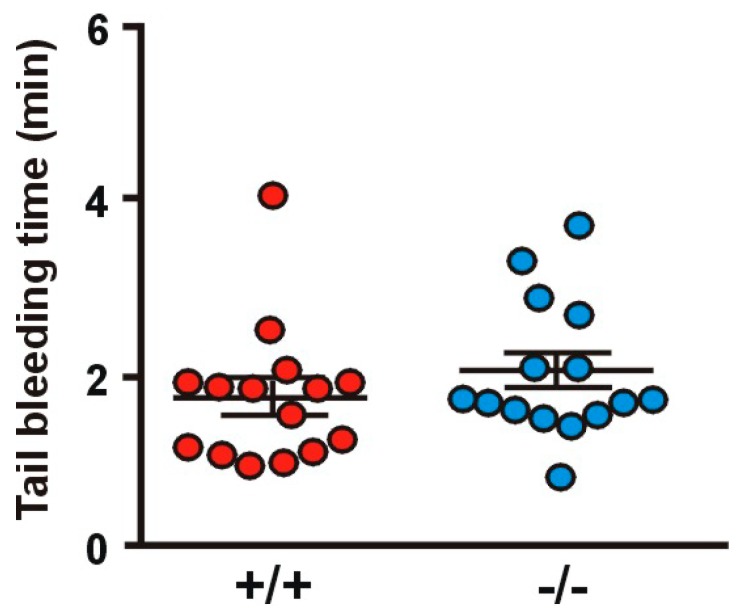
Tail bleeding time. Tests were performed by cutting off 2 mm of the tail tip and immediately placing the tail in PBS at 37 °C. The time until hemostasis was recorded for up to 10 min and re-bleeding monitored for 60 s beyond hemostasis. Data represent mean ± SEM of 15 animals. No significant differences were found between WT and KO (Student’s *t*-test).

To evaluate the influence of *Coro1a* deletion on hemostasis, we examined tail bleeding (Figure 8). Both Coro1 KO and WT animals showed a comparable average bleeding time (1.99 ± 0.19 min in the KO vs. 1.71 ± 0.20 min in the WT). In these experiments, two WT and two KO mice out of 15 per genotype re-bled within one minute of cessation of bleeding.

## 3. Discussion

The availability of animal models has significantly contributed to elucidate the roles in platelet function of cytoskeleton proteins, which usually cannot be targeted pharmacologically [30]. Here we present a functional characterization of Coro1, an abundant class I coronin, in a KO mouse model. The salient phenotype of Coro1 deficient platelets is the impaired translocation of integrin β2 to the cell surface upon thrombin stimulation, in the absence of any alteration in a range of morphological and functional tests. Our study broadly confirms a recent report by Stocker et al. and explores aspects not covered there [20]. However, we failed to observe some of the mild defects reported by Stocker et al., namely increased relative platelet size and adhesion receptor expression, decreased platelet spreading area upon stimulation with thrombin or collagen, and decreased velocity of aggregation in response to low-dose collagen [20]. These divergent outcomes could be tracked back to methodological differences, size of experimental populations, and statistical analysis. For example, Stocker et al. used an impedance-based method on whole blood to study aggregometry, whereas we used light transmission aggregometry on washed platelets. Impedance-based aggregometry on whole blood captures responses that depend on interactions with leukocytes and red blood cells and is, therefore, closer to the physiological situation, however, it is considered insensitive to low levels of platelet activation. Light transmission aggregometry, by contrast, requires more manipulations but takes platelets at face value, without the influence of variations in hematocrit and cellular content [31,32]. The different methods might have influenced platelet reactivity, causing opposite outcomes, although the differences between WT and KO were always small.

While translocation and activation of integrin αIIbβ3 were not affected in the Coro1 KO platelets, we observed impaired translocation of integrin β2 by deletion of Coro1, suggesting that Coro1 is specifically implicated in the regulation of this integrin in platelets. Integrin β2, a component of lymphocyte function-associated antigen 1 (LFA-1) when associated with integrin αL, is one of the 6 integrins expressed in mouse platelets and the fourth most abundant [25]. β2 integrins are important for polymorphonuclear neutrophil adhesion to the endothelium and subsequent events, like extravasation [33]. Coro1 is critical for these processes because it interacts with the cytoplasmic tail of integrin β2 and regulates the accumulation of activated integrin in focal zones of adherent cells [10]. In platelets, LFA-1 has not been extensively investigated. Platelets from mice deficient in integrin β2 are characterized by a shorter lifespan, reduced adhesion to the endothelium in response to tumor necrosis factor (TNF), and caspase activation [24]. Stocker et al. reported a normal lifespan of Coro1 deficient platelets, suggesting that this coronin is not the only protein responsible for the regulation of integrin β2 [20]. Similarly, we did not observe any defective adhesion and spreading on an ICAM-1 surrogate matrix, suggesting that Coro1 deficient platelets retain sufficient binding capacity through LFA-1 and/or other mechanisms. In line with this observation, Stocker et al. reported unaffected accumulation of neutrophils within arterial thrombi in Coro1 deficient platelets [20]. However, the role of integrin β2 in platelet–leukocyte interaction is difficult to dissect due to concurrent and more prevalent mechanisms mediating those interactions [34] and to the fact that the interactions mediated by LFA-1 and ICAM family molecules are reciprocal: both are present simultaneously in platelets and leukocytes. A rigorous attempt at exploring this aspect would require the generation of a platelet-specific Coro1 knockout model combined with platelet-specific deletion of ICAM-2, the adhesion molecule isoform present in the platelet membrane [25].

Jayachandran et al. have uncovered the role of Coro1 in modulating the cAMP signaling pathway in excitatory neurons, where deficiency of the protein resulted in the loss of excitatory synapses and a range of neurobehavioral disabilities [9]. Coro1 interacts with Gαs in a stimulus-dependent manner, leading to increased cAMP production [9]. Moreover, the association of Coro1 with Gαs is regulated by cyclin-dependent kinase 5 (CDK5)-mediated phosphorylation of Coro1 on two particular threonine residues [35]. Furthermore, Coro1 regulates cAMP signaling in T cells [29] whereas the homolog in *Dictyostelium discoideum* regulates cAMP-dependent initiation of multicellular aggregation [36] and the homolog in the fungus *Magnaporthe oryzae* interacts with a Gαs subunit to regulate cAMP production and pathogenicity [37]. In platelets, Gαs activation and subsequent cAMP production are coupled to binding of PGI2 to its G protein-coupled receptor. Although Coro1 is able to co-immunoprecipitate Gαs in platelets [6], absence of Coro1 does not appear to be detrimental to the production of cAMP, as demonstrated by the ability of Coro1 deficient platelets to phosphorylate VASP and block the effects of thrombin stimulation when exposed to PGI2. The role of Coro1 in cAMP regulation is, however, complex. In T cells depletion of Coro1 results in reduced production of cAMP, however, cAMP levels are increased due to a compensatory decrease in phosphodiesterase 4 (PDE4) levels [29]. Further research would be needed to clarify whether Coro1 regulates the cAMP pathway in platelets and, if so, through which molecular mechanisms. PDE4 is absent [38] but CDK5 is present both in human and mouse platelets [25,39], although the role of the latter in platelets has not been addressed so far. In addition, we have reported the presence of Coro2 and Coro3 in immunocomplexes with Gαs [6], suggesting that these two class I coronins may compensate for the absence of Coro1 for regulation of the cAMP pathway. Functional compensation by other class I coronins might also explain the retained ability of the Arp2/3 complex to accumulate at the cell cortex and enable the formation of lamellipodia and consequently spreading. We have shown that Coro2 and 3 can be found in immunocomplexes with ARPC2 and accumulate at the cell cortex of spread platelets [6].

In summary, we propose that class I coronins display a large extent of functional overlap in platelets. This would explain the absence of a strong phenotype in most platelet functional assays while aspects like integrin β2 translocation reported by us and the formation of F-actin and cofilin dephosphorylation in response to agonists reported by others [20] are specifically or more strongly dependent on Coro1 function. This is not uncommon among components of the actin cytoskeleton, where examples abound [30]. Thus, disruption of the Arp2/3 complex regulators cortactin and its homolog HS1 does not cause any noticeable alteration in platelet function, indicating that their roles might be fulfilled by other proteins [40]. Similarly, disruption of the formin mDia results in no major platelet phenotype, pointing at functional compensation by other formins present in platelets [41]. Future studies toward the elucidation of coronin function in platelets will, therefore, require the generation of mouse models lacking two or three class I coronins in order to arrive at a complete picture of the shared and unique roles of these proteins.

## 4. Materials and Methods

### 4.1. Reagents

Primary antibodies against following proteins were used: Coro1 (ab56820 and ab72212), β-actin (ab20272) from Abcam (Cambridge, UK); Coro3 (K6-444 hybridoma supernatant) [42]; β3-integrin (HC93 sc-14009) and Gαs (sc-823) from Santa Cruz Biotechnology (Heidelberg, Germany); phosphor-VASP(Ser157) (#3111) from Cell Signaling Technology (Leiden, The Netherlands); GAPDH (6C5-CB1001) from Calbiochem/Merck (Watford, UK); p34-Arc/ARPC2 (07-227) from Millipore/Merck. Secondary antibodies Alexa Fluor 568-conjugated anti-rat or anti-rabbit immunoglobulins (Molecular Probes, Thermo Fisher Scientific, Altrincham, UK) were used for immunofluorescence. IRDye 680 or IRDye 800 anti-mouse and anti-rabbit immunoglobulins (LI-COR Biosciences, Lincoln, NE, USA) were used for Western blot. Human fibrinogen was from Enzyme Research (Swansea, UK), collagen (Kollagenreagens Horm) was from Takeda (Osaka, Japan), and PGI2 was from Cayman Chemical (Ann Arbor, MI, USA). Phosflow Lyse/Fix Buffer and P-selectin were from BD Biosciences (Oxford, UK). Gly-Phe-Hyp-Gly-Glu-Arg (GFOGER) and collagen-related peptide (CRP) were from Cambridge University (Cambridge, UK). U46619 was from Enzo (Exeter, UK). CK666 was from Tocris Bioscience (Abingdon, UK). Thrombin, ADP and FITC, or TRITC-conjugated phalloidin were from Merck (Dorset, UK). Other reagents were from Merck unless otherwise indicated.

### 4.2. Experimental Animals

C57Bl/6 mice with a homozygous targeting of the *Coro1a* gene have been previously described [15] and are available from The Jackson Laboratory (JAX stock no. 030203). The animals were kept in the animal facility of the University of Hull using standard conditions. All animal work was performed in accordance with UK Home Office regulations, UK Animals (Scientific Procedures) Act of 1986, under the Home Office project license no. PPL 70/8253 (2 January 2015). Age-matched WT littermates were used as controls in all experiments. Twelve to twenty-week-old animals were used for experiments.

### 4.3. Mouse Platelet Preparation

Blood was taken by cardiac puncture into acid citrate dextrose (ACD) (29.9 mM trisodium citrate, 113.8 mM glucose, 72.6 mM NaCl, and 2.9 mM citric acid, pH 6.4) or sodium citrate (109 mM tri-sodium citrate pH 7.4) and centrifuged at 100× *g* for 5 min. The platelet-rich plasma (PRP) was collected in a separate tube, modified Tyrode’s buffer was added to the pellet, and the procedure repeated to increase the platelet yield. For washed platelet preparation, the PRP was pelleted at 800× *g* for 6 min and platelets resuspended in modified Tyrode’s buffer and allowed to rest for 30 min at 37 °C prior to experiments.

### 4.4. Western Blot

Lysates were prepared from washed platelet suspensions by mixing with one volume of 2× Laemmli buffer. Proteins were resolved by SDS-polyacrylamide gel electrophoresis (PAGE) and blotted onto polyvinylidene difluoride (PVDF) membrane. The membrane was incubated with the relevant primary antibody and the corresponding fluorochrome-labeled secondary antibody and visualized and quantified with an LI-COR Odyssey CLx Imaging System (LI-COR Biosciences).

### 4.5. Flow Cytometry

PRP was prepared in sodium citrate and stimulated with thrombin (in the presence of 10 µM Gly-Pro-Arg-Pro-NH_2_), CRP, ADP, or U46619 for 20 min at 37 °C in the presence of FITC-conjugated anti-P-selectin (BD Biosciences), PE-conjugated JON/A (Emfret, Würzburg, Germany) and APC/Cy7-conjugated anti-CD63 (Biolegend) antibodies. Platelets were subsequently fixed and analyzed by fluorescence-activated cell sorting (FACS) using an LSRFortessa cell analyzer (BD Biosciences) and FlowJo software.

For receptor expression studies, PRP was incubated with FITC-conjugated antibodies directed against surface membrane glycoproteins GP1b (CD42b), GPVI, integrin α2 (CD49b) (Emfret, Eibelstadt, Germany), integrin αIIb (CD41) (BD Biosciences, Oxford, UK), or PE-conjugated antibodies against integrin β2 (CD18) (Biolegend). Receptor expression was also studied upon stimulation with 0.1 U/mL thrombin for 20 min at 37 °C in the presence of 10 µM Gly-Pro-Arg-Pro-NH_2_. Platelets were subsequently analyzed by FACS.

### 4.6. Aggregation, Spreading, and Immunostaining

Platelet aggregation in response to agonists was recorded in washed platelets under constant stirring conditions (1000 rpm) for 7 min at 37 °C using light transmission aggregometry with a CHRONO-LOG 490 aggregometer (CHRONO-LOG, Havertown, PA, USA). Washed platelets in suspension were fixed with an equal volume of ice-cold 4% paraformaldehyde (PFA) and spun at 350× *g* for 10 min on poly-l-lysine (0.01% in PBS) coated coverslips. Platelets were stained for 1 h at room temperature with the indicated primary antibodies followed by the corresponding secondary antibodies and fluorescently labeled phalloidin diluted in PBG (0.5% bovine serum albumin (BSA), 0.05% fish gelatin in PBS). For adhesion studies, coverslips were coated overnight at 4 °C with fibrinogen, collagen, CRP, or GFOGER in PBS at the concentrations indicated and blocked with 5 mg/mL heat-denatured fatty acid free BSA for 1 h before the experiment. For adhesion on native BSA, coverslips were coated overnight at 4 °C with 5 mg/mL fatty acid free BSA in 0.05 M sodium bicarbonate buffer pH 9 [27]. Washed platelets were allowed to spread for 45 min at 37 °C, fixed with 4% PFA for 10 min, permeabilized with 0.3% Triton X-100 for 5 min, and stained as described above for platelets in suspension. Platelets were imaged by fluorescence microscopy using a Zeiss ApoTome.2 equipped with an AxioCam 506 and Zeiss Plan-Apochromat 63× and 100× NA 1.4 objectives. Platelets were manually counted, and the surface coverage area was analyzed by thresholding using ImageJ.

### 4.7. Tail Bleeding Assay

Mice were anesthetized with 50 mg/kg ketamine and 1 mg/kg medetomidine. The tail was cut off at 2 mm from the tip and immediately immersed in 37 °C PBS. Bleeding time between the cut and cessation of bleeding for at least one minute was monitored by visual inspection until hemostasis for up to 10 min.

### 4.8. Statistical Analysis

Experimental data were analyzed by GraphPad Prism v6.0 (La Jolla, CA, USA). Data are presented as mean ± standard error of the mean (SEM) of at least 4 independent experiments. Normality was assessed by the Shapiro–Wilk test. Differences between groups were assessed using the appropriate parametric or nonparametric test and statistical significance was taken at *p* ≤ 0.05. 

## Supplementary Materials

The following are available online at https://www.mdpi.com/1422-0067/21/1/356/s1, Figure S1: Full length blots corresponding to Figure 1A; Figure S2: Full length blots corresponding to Figure 7A. The fourth lane of each genotype corresponds to an experimental condition not included in the article.

## Abbreviations

ACD	acid citrate dextrose
ADP	adenosine diphosphate
ARPC2	p34-Arc, component of the Arp2/3 complex
BSA	bovine serum albumin
cAMP	cyclic adenosine monophosphate
CDK5	cyclin-dependent kinase 5
CDx	cluster of differentiation x
CRP	collagen related peptide
FACS	fluorescence-activated cell sorting
FITC	fluorescein isothiocyanate
GEFOGER	Gly-Phe-Hyp-Gly-Glu-Arg
ICAM-1	intercellular adhesion molecule 1
KO	knockout
LFA-1	lymphocyte function-associated antigen 1
PAGE	polyacrylamide gel electrophoresis
PBS	phosphate buffered saline
PDE4	phosphodiesterase 4
PFA	paraformaldehyde
PGI2	prostacyclin
PRP	platelet-rich plasma
SEM	standard error of the mean
TNF	tumor necrosis factor
TRITC	tetramethylrhodamine isothiocyanate
VASP	vasodilator-stimulated phosphoprotein
WT	wild type
