# Supplementary material for: Coronin 1 Is Required for Integrin β2 Translocation in Platelets
Source: Int J Mol Sci. 2020 Jan 5;21(1):356. doi: 10.3390/ijms21010356 (PMC6982036; doi:10.3390/ijms21010356)
Supplement: Supplementary file 1 [file ijms-21-00356-s001.pdf]

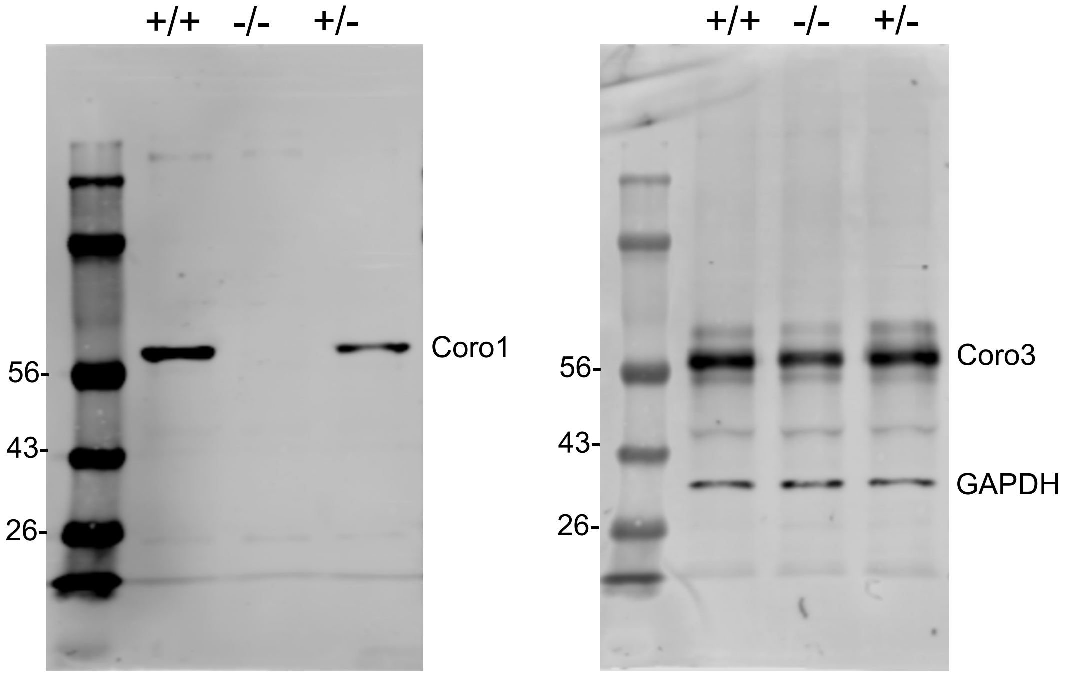

Supplemental Figure 1. Full length blots corresponding to Figure 1A.

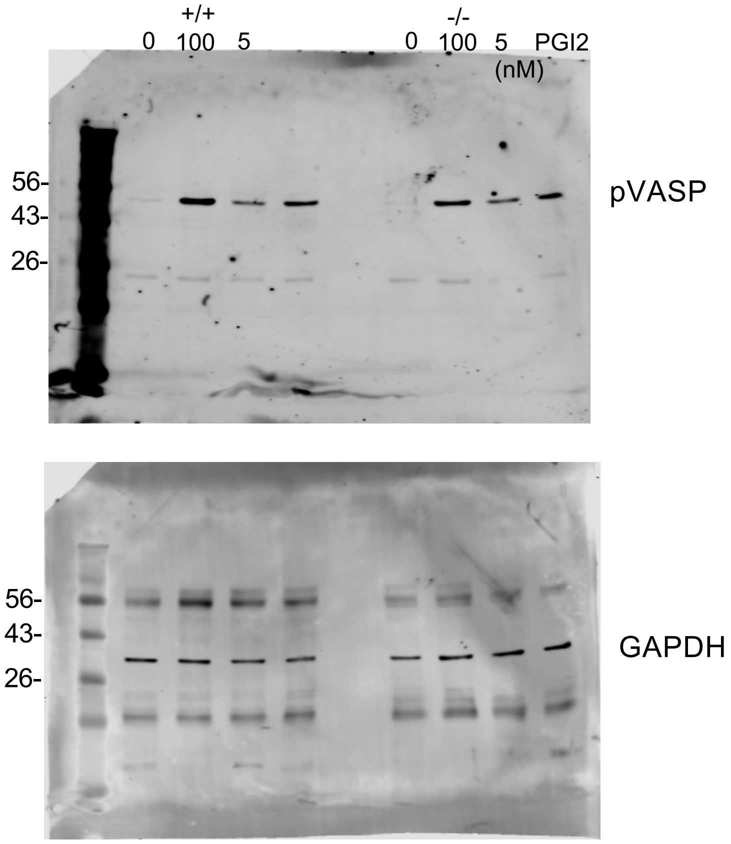

Supplemental Figure 2. Full length blots corresponding to Figure 7A. The fourth lane of each genotype corresponds to an experimental condition not included in the article.
